# Variants in the zinc transporter-3 encoding gene (SLC30A3) in schizophrenia and bipolar disorder: Effects on brain glutamate–A pilot study

**DOI:** 10.3389/fpsyt.2022.929306

**Published:** 2022-09-20

**Authors:** Luke A. Jelen, Mark S. Green, Sinead King, Alex G. Morris, Xinyuan Zhang, David J. Lythgoe, Allan H. Young, Jacqueline De Belleroche, James M. Stone

**Affiliations:** ^1^Institute of Psychiatry, Psychology and Neuroscience, King's College London, London, United Kingdom; ^2^South London and Maudsley NHS Foundation Trust, Bethlem Royal Hospital, Beckenham, United Kingdom; ^3^Department of Psychiatry and Behavioural Sciences, Stanford University, Palo Alto, CA, United States; ^4^Centre for Neuroimaging and Cognitive Genomics, National University of Ireland, Galway, Ireland; ^5^Imperial College London, Hammersmith Hospital Campus, London, United Kingdom; ^6^Psychiatry, Department of Neuroscience, Brighton and Sussex Medical School (BSMS), University of Sussex, Brighton, United Kingdom; ^7^Department of Psychiatry, Sussex Partnership Foundation Trust, Eastbourne DGH, Eastbourne, United Kingdom

**Keywords:** glutamate, ZnT3, zinc transporter, MRS-1H nuclear magnetic resonance spectra, schizophrenia, bipolar (affective/mood) disorders

## Abstract

Zinc transporter 3 (ZnT3) has been implicated in the aetiopathology of schizophrenia. In this pilot study, we tested the hypothesis that the presence of a minor allele of two variants in the gene encoding ZnT3 (SLC30A3) affects brain glutamate and cognitive activity in patients with schizophrenia and bipolar affective disorder. Fifteen patients with schizophrenia (SCZ), 15 with bipolar affective disorder type 2 (BD), and 14 healthy volunteers (HV) were genotyped for two SLC30A3 single nucleotide polymorphisms (rs11126936 and rs11126929). They also underwent structural and functional MRI (n-back) imaging as well as static (PRESS) and functional magnetic resonance spectroscopy (n-back) on a 3 Tesla MRI system. SCZ with at least one copy of the minor allele showed reductions in dorsal anterior cingulate cortex glutamate during the n-back task, whereas SCZ without the minor allele showed an increase in glutamate. BD with the minor allele had reduced glutamate in the anterior cingulate cortex (*p* < 0.05). There was no effect of SLC30A3 genotype on BOLD activation during n-back or on cortical brain volume. This study supports the further investigation of SLC30A3 and its role in glutamatergic neurotransmission and in the neuropathology of mental illness.

## Introduction

Zinc, an essential trace element required for a variety of cellular processes including DNA replication, transcription and protein synthesis, has crucial roles in endocrine, immunological, neural and cognitive functioning (1). Increasing evidence from clinical, molecular and genetic studies implicate zinc dysregulation and deficiency in the pathophysiology of a range of neuropsychiatric conditions including schizophrenia and mood disorders (2, 3).

Zinc is prevalent in its ionic form (Zn^2+^) in glutamatergic nerve terminals in regions of the brain involved in emotion, learning and memory including the frontal cortex, amygdala and hippocampus (4, 5). Zn^2+^ is released upon neuronal activation and interacts with a number of receptors including NMDA (N-methyl-D-aspartate) and GABA_A_ (γ-aminobutyric acid type A) receptors, acting as a neuromodulator to control synaptic excitability (6).

As Zn^2+^ is also neurotoxic, neurons have a variety of homeostatic mechanisms in place to maintain extracellular and intracellular levels at non-toxic concentrations (5). These processes are supported by a diverse family of dedicated zinc transporter proteins that control the uptake, efflux, and compartmentalisation of zinc (7). One of these zinc transporter proteins, encoded by the SLC39A13 gene, transports zinc into the cytoplasm. Novel and possibly damaging mutations of this gene have been reported in five schizophrenic patients, where the effect of one such mutation was examined using MRI, which found reduced zinc concentration in the rostral anterior cingulate cortex associated with reduced verbal intelligence and negative symptoms (3). A second member of this set of proteins, the zinc transporter 3 (ZnT3) is the sole mechanism responsible for concentrating Zn^2+^ within synaptic vesicles of glutamatergic terminals (8). A single nucleotide polymorphism (SNP) in the SLC30A3 gene, which encodes for the ZnT3 protein, (rs11126936) has been associated with verbal learning deficits (9). Recent work has highlighted the role of ZnT3 in controlling gain in auditory circuits *via* parvalbumin cells (10), and in discriminating between different auditory signals, a function of particular significance to the sensory processing deficits in patients with schizophrenia (11). Using mouse models of autism spectrum disorder, the drug clioquinol was found to mobilize the transynaptic source of Zn^2+^ that requires the ZnT3 transporter. This rescued social interaction *via* postsynaptic activation of Src family Kinase and NMDA receptors (12).

A post-mortem study of gene expression in two separate cohorts of patients with schizophrenia revealed reduced transcripts from the SLC30A3 gene, in the prefrontal cortex: Brodmann area (BA) 10 (Charing Cross Hospital Prospective Collection) and BA 9 (Harvard Brain Bank) (13). Lowered zinc levels in serum and brain have been reported in schizophrenia subjects, where dysregulation of zinc homeostasis in schizophrenia is also reflected by increased expression levels of SCL39A12 in BA9 (3), in contrast to the lowered levels of vesicular SLC30A3 noted above. The mechanisms underlying these contrasting changes, along with the effect of SNPs, remain to be elucidated.

Subsequent work on variants in the SLC30A3 gene using a UK case-control cohort revealed that the minor alleles of four SNPs, including rs11126936 and rs11126929, were associated with schizophrenia in female patients (14, 15). A meta-analysis of 14 samples of European descent (total *N* = 18,826), including data from genome-wide association studies (GWAS), confirmed the associations of rs11126936 and rs11126929, where the minor alleles were again significantly overrepresented in females with schizophrenia (14). In contrast, a polymorphism of the SLC30A3 gene (rs11126936) has been shown to be associated with major depressive disorder in a cohort of Asian participants, with those homozygous for the minor allele having reduced risk of MDD (16).

In this pilot study we investigated the *in vivo* relationship between allelic variants of the SLC30A3 gene and (a) brain structure and volume, measured using structural magnetic resonance imaging (MRI), (b) brain function during the n-back task, measured using functional magnetic resonance imaging (fMRI), (c) glutamatergic neurochemistry, measured using proton magnetic resonance spectroscopy (^1^H-MRS) and (d) glutamate dynamics during the n-back task, measured using proton functional magnetic resonance spectroscopy (^1^H-fMRS), in groups of patients with schizophrenia and bipolar II disorder together with healthy volunteers.

## Materials and methods

### Participants

Fifteen participants with a diagnosis of schizophrenia (SCZ) and fifteen participants with bipolar affective disorder type 2 (BD) were compared with fourteen healthy volunteers (HV). Recruitment procedures were as previously described (17, 18). Thirteen schizophrenia patients were receiving regular antipsychotic medication (four taking olanzapine; three taking risperidone; two taking aripiprazole; three taking clozapine; one taking haloperidol) and two were not currently medicated. Five BPII patients were receiving antidepressants (three taking citalopram; one taking sertraline; one taking fluoxetine) and the remaining ten were not taking psychotropic medication. The healthy controls were medication-naïve. Demographic details are summarized in Table 1. The data were collected as part of a study approved by the London Harrow Research Ethics Committee and all participants provided written informed consent to participate.

**Table 1:** Participant demographics.

**Group**	**SCZ**	**BD**	**HV**	***P-*value**
*n*	15	15	14	
Age	40.1 ± 10.0	38.6 ± 10.6	33.8 ± 10.5	0.248
M/F	11/4	7/8	7/7	0.191

### Genotyping

Genomic DNA was extracted from whole blood using the Paxgene Blood DNA Kit as described in the protocol (PreAnanytiX Company, CH-8634 Hombrechtikon, Switzerland) and stored at −20°C. Genotyping was performed on two SNPs in SLC30A3 (rs11126936 with major and minor alleles G/T respectively and rs11126929, A/G). These SNPs were in a haplotype block with r^2^ = 1 (13) and selected because they were previously found to be associated with schizophrenia by a meta-analysis of a series of 14 European samples consisting of 10,802 controls and 8,024 schizophrenia cases (14). The region containing rs11126929 is a potential binding site for POLR2A and rs11126936 had the highest RegulomeDB score likely to affect the upstream gene ATRAID (14). Genotyping was carried out using the KASP^TM^ assay method (Kompetitive Allele Specific PCR). This involved allele-specific PCR in the initial round of PCR followed by either HEX or FAM labeled tail extension and detection of each allele in subsequent rounds of PCR. This was carried out by LGC laboratories (Units 1-2, Trident Industrial Estate, Hoddesdon, Hertfordshire, EN11 0WZ). Participants were divided into two groups according to genotype for each SNP: Major allele genotype is homozygous for reference allele; Minor allele genotype is heterozygous or homozygous for the alternate allele. The statistical tests were carried out in gPlink (https://zzz.bwh.harvard.edu/plink/gplink.shtml).

### Structural data acquisition

Structural MRI, fMRI and proton spectroscopy data were obtained in a single session for each participant using one of two GE Discovery MR750 model 3-Telsa scanners. Scanning took place at the Centre for Neuroimaging Sciences (CNS). Both scanners were fitted with a body transmitter coil and 12-channel head receiver coil. For the structural data, a high resolution T1-weighted 3D MPRAGE sequence was used (TR = 7.31 ms, TE = 3.02 ms, TI = 400 ms, FOV = 270 mm, flip-angle (α) = 11°, matrix size = 256 × 256 mm^2^, slice thickness = 1.2 mm, 196 slices).

### Freesurfer preprocessing and analysis

Images were preprocessed by re-slicing to be compatible with the Freesurfer v6.0.0 package (19). Subsequent preprocessing was carried out *via* the recon-all pipeline in Freesurfer using a smoothing kernel of 25 mm and a different onset different slope (DODS) design in which six categories (one for every combination of diagnostic group and genotype) and a covariate, age, was modeled. Subsequently, left- and right-hemisphere volumetric measurements were extracted from the Desikan-Killiany aparc atlas (20). At the second-level mean ROI volume was entered into a 2x3 ANOVA with factors of genotype and diagnosis. We controlled Type I error rate using the false detection rate (FDR) Benjamini-Hochberg method (21). Subsequently, for ROIs where diagnosis proved significant, planned (two-tailed) two-sample *t*-test *post-hoc* testing was performed. These analyses were performed using R routines embedded in python (3.7) scripts.

### N-back task paradigm

For the fMRI n-back task, participants were exposed to 0-back, 1-back, 2-back and 3-back blocks. As sequential letters were presented individually, participants were asked to respond as quickly and accurately as possible each time a letter was presented that was the same as one presented “n” items before. Twelve blocks were presented in a pseudorandomised order and lasted for 28s each. Each block contained 14 sequential 2s visual presentations of letter stimuli. Blocks were preceded by 3s visual indicators of block n-value.

A similar design was used for the ^1^H-fMRS n-back task. Here, 18 alternating blocks lasted for 48s each with 18 stimuli presented within each block. Stimuli were presented for 1s, with 1.5s between stimuli and participants had 1.9s to respond (17).

### FMRI N-back data acquisition

A gradient-echo, echo-planar imaging T2^*^-weighted sequence was used to image the BOLD contrast [Echo Time (TE) = 30 milliseconds (ms), Recovery Time (TR) = 2,000 ms, flip angle = 75°, matrix size = 64 x 64, field of view = 240 x 240 millimeters (mm), voxel size = 3.75 x 3.75 x 3.75 mm]. For each participant 189 volumes were acquired. These volumes consisted of 41 near axial slices oriented parallel to the anterior commissure-posterior commissure (AC-PC) line. Slices were acquired in descending order. For one of the SCZ participants no functional data was obtained.

### FMRI N-back preprocessing and analysis

Preprocessing and analysis of fMRI data was performed in SPM12 (http://www.fil.ion.ucl.ac.uk/spm/software/spm12/). Following resetting the origin to the anterior commissure, functional volumes were slice time corrected to a medical reference slice and spatial realignment was performed. A resliced mean functional image was generated and co-registered to the participant's MPRAGE image prior to normalization to the standard Montreal Neurological Institute (MNI) space. Volumes were smoothed using a Guassian filter set to 8 mm at FWHM. Quality assurance performed following preprocessing included checking functional volumes for coregistration to the participants structural image and graphing of realignment parameters.

**Figure 1:**
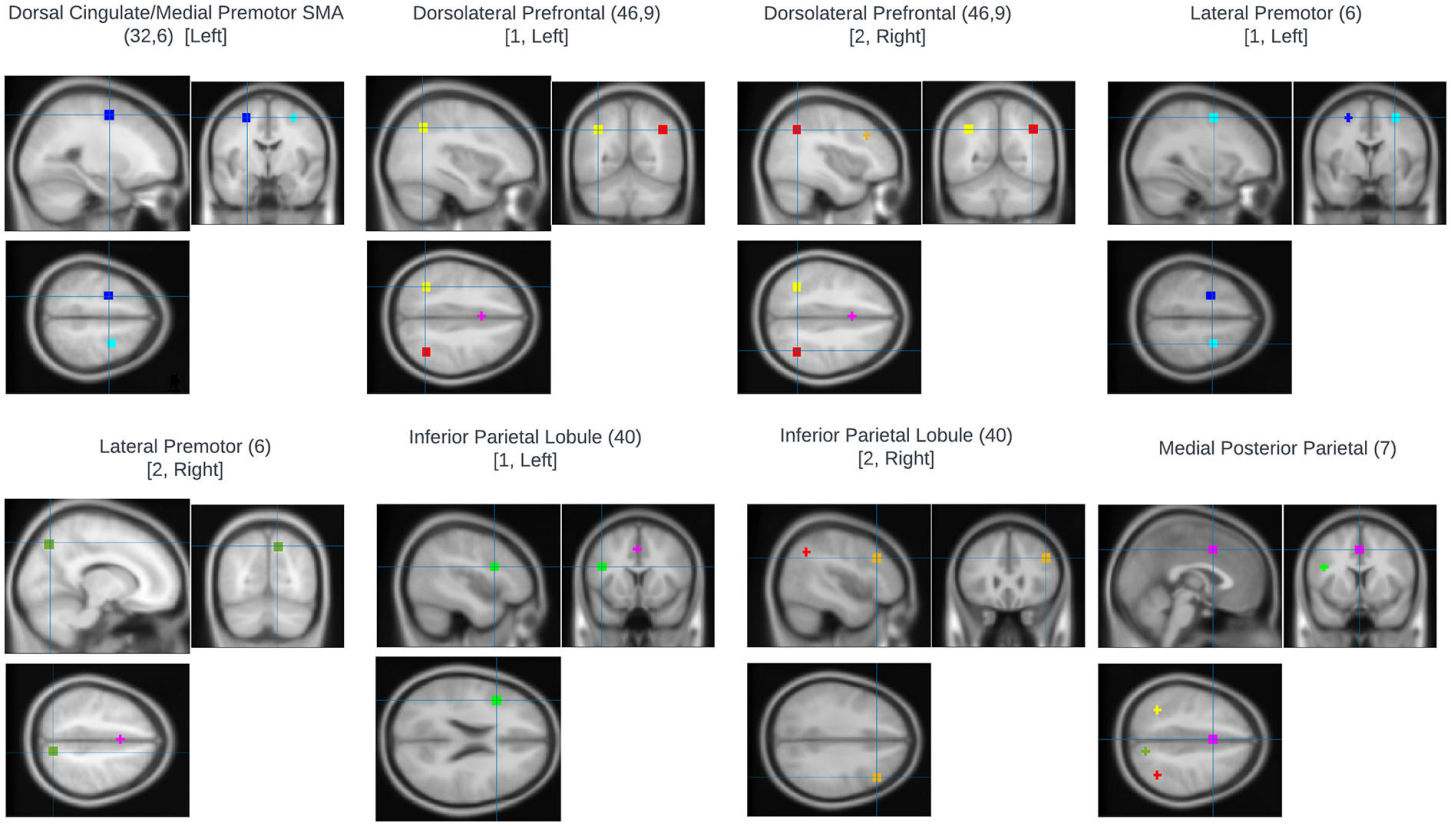
Regions of interest used for fMRI analysis. All were based on the prior meta-analysis of the n-back carried by Owen et al. (23). Sphere centrum coordinates were selected for each meta-analytic peak associated with an Activation Likelihood Estimate (ALE) score ≥0.04.

An ROI approach using loci representative of the n-back network was taken using MarsBaR (22). Eight ROIs were defined based on the prior meta-analysis of the n-back carried by Owen et al. (23). Sphere centrum coordinates were selected for each meta-analytic peak associated with an Activation Likelihood Estimate (ALE) score ≥0.04. Talairach coordinates were converted to MNI space using the inverse mni2tal transform and spheres of 6 mm radius were defined for each ROI (Table 2; Figure 1). Masks were created in FSL and visualized in MRIcron. For all ROIs, mean voxel-wise 2-back>0-back contrast values were computed for each participant.

**Table 2:** MNI coordinates for fMRI regions of interest.

		**MNI**		
**ROI**	**X**	**Y**	**Z**	**ALE**
Dorsal cingulate/medial premotor (SMA) (32, 6) [Left]	−26.26	−2.68	56.45	0.0488
Dorsolateral prefrontal (46, 9) [1, Right]	40.40	−51.38	38.64	0.0535
Dorsolateral prefrontal (46, 9) [2, Left]	−44.44	17.40	24.86	0.0422
Inferior parietal lobule (40) [1, Right]	10.10	−70.43	48.52	0.0555
Inferior parietal lobule (40) [2, Left]	−36.36	−53.54	40.70	0.0551
Lateral premotor (6) [1, Right]	28.28	1.54	54.49	0.0684
Lateral premotor (6) [2, Right]	40.40	31.40	34.31	0.0515
Medial posterior parietal (7)	−2.02	10.19	46.24	0.06

MNI coordinates for ROI centers of mass, with accompanying ALE scores from Owen et al. (23). ROIs were modeled as 6 mm spheres.

Mean signal change was entered into a (2 x 3) factorial MANOVA with factors genotype and diagnostic group, followed by a (2 x 3) ANOVA identical to that used in the Freesurfer analysis. Type I error-rate inflation was controlled for using the Holm-Sidak method (24).

### ^1^H-MRS data acquisition and analysis

^1^H-MRS data were acquired as previously reported (18). ^1^H-MRS data were collected from a voxel located in the bilateral anterior cingulate cortex (ACC) (20 x 20 x 20 mm) using each participant's T1 weighted MPRAGE image for voxel definition. A single spectrum was obtained using point resolved spectroscopy (PRESS) acquisition (TE = 30 ms, TR = 3,000 ms, 96 averages). LCModel was used to analyse all spectra and generate water-scaled metabolite levels (25). T1-weighted images were segmented into gray matter, white matter and cerebrospinal fluid (CSF) compartments using SPM and in-house software was used to determine CSF, gray- and white- matter content in the spectroscopy voxel. Metabolite levels were corrected for the presence of CSF in the voxel, taking into account the default CSF, gray matter and white matter water concentrations employed by LCModel, using the formula Mcorr = M^*^(1.207^*^gm + wm + 1.548 CSF)/(1–CSF), where M is the uncorrected metabolite value and wm, gm and CSF are the white and gray matter and CSF fractions for the spectroscopy voxel. Any poorly fit metabolite measures [estimated Cramer–Rao minimum variance bounds (CRMVB) > 20%] were excluded from further analysis. Two-way analyses of variance (ANOVA) were used to examine the influence of genotype and group on Glu and Glx levels with Sidak's *post-hoc* tests for multiple comparisons.

### ^1^H-FMRS N-back data acquisition and analysis

^1^H-fMRS data were acquired as previously reported (17). The ^1^H-fMRS voxel (30 x 20 x 20 mm) was aligned with its inferior edge on the superior surface of the corpus callosum, with its center point positioned 7 mm posterior to the genu of the corpus callosum in the sagittal plane and midline of the brain using each participant's T1-weighted MPRAGE image. PRESS (TR = 2,000 ms, TE = 105 ms, NEX = 8) was used to acquire ^1^H-fMRS spectra individually throughout the n-back task. 16 water-unsuppressed and 432 water-suppressed spectra were collected for each participant. During each of the eighteen 48s n-back blocks, 3 individual spectra were acquired (one every 16s, NEX = 8).

The TARQUIN software package, version 4.3.6 (26), was used to perform spectral analysis which performed a fully automated fit to the data using a predefined basis set (alanine; aspartate; creatine (Cr); gamma-aminobutyric acid; glucose; glutamine; glutathione; glutamate (Glu); glycerophosphorylcholine; myo- inositol; lactate; lipid peaks at 0.9, 1.3a, 1.3b, and 2.0 ppm; macromolecules at 0.9, 1.2, 1.4, and 2.0 ppm; N-acetyl-aspartate (NAA); N-acetyl-aspartate glutamate; phosphorylcholine; phosphocreatine (PCr); scyllo-inositol; and taurine.

The spectra acquired during the 0-back and 2-back conditions (8 blocks of each) were averaged together and measures of Glu and Glx (Glu + glutamine), scaled to total creatine (TCr: Cr + PCr) were recorded. TCr scaling was used to address potential line width reductions in spectral measures that can be expected due to reductions in local field homogeneity that accompanies the BOLD effect (27, 28). Any metabolite measures with a CRMVB > 20%, a signal-to-noise ratio (SNR) < 5, a full-width half- maximum (FWHM) of > 0.10 ppm or fit quality (Q) of > 2.5 were excluded.

For the ^1^H-fMRS data, 2x2 repeated-measures analyses of variance (rmANOVA) were applied, using Glu/TCr and Glx/TCr levels across averaged 0-back and 2-back task conditions, with genotype as a between-subjects factor to examine the effect of genotype in each group individually.

## Results

### SLC30A3 genotype

The results of the genotyping analysis are shown in Table 3. In each participant, the minor allele frequency of the SNP rs11126929 was identical to that found in rs11126936 owing to strong linkage disequilibrium between them. The SNP genotypes with the minor alleles showed an association both with BD and SCZ (*p* < 0.05 FDR).

**Table 3:** SLC30A3 genotype for rs11126929 A/G and rs11126936 SNPs by group (HV, healthy volunteers; SCZ, patients with schizophrenia; BD, patients with bipolar affective disorder type 2).

	**rs11126929 A/G**		**rs11126936 G/T**	
	**Major (A:A)**	**Minor (A:G or G:G)**	**Major (G:G)**	**Minor (G:T or T:T)**
HV	10 (76.9%)	3 (23.1%)	10 (76.9%)	3 (23.1%)
SCZ	6 (40.0%)	9 (60.0%)	6 (40.0%)	9 (60.0%)
BD	5 (33.3%)	10 (66.7%)	5 (33.3%)	10 (66.7%)

### Structural MRI

There was a significant difference between the three groups in 17 brain regions, corrected for FDR, with HV having significantly larger volumes than SCZ in all 17 regions, and BD having significantly larger volumes than SCZ in 6 of these regions (*p* < 0.05; Table 4). There was no overall effect of genotype on brain volumes.

**Table 4:** Volumetric brain regions showing significant differences by diagnostic group.

**ROI**	***F*-test**	**Direction**	***T*-test**
lh_entorhinal_volume	*F*_(2.0, 36.0)_ = 2.874, *p* = 0.004	HV > SCZ	*t*_(25.0)_ = 3.288, *p* = 0.009
lh_inferiorparietal_volume	*F*_(2.0, 36.0)_ = 0.115, *p* = 0.036	HV > SCZ	*t*_(27.0)_ = 3.284, *p* = 0.009
		BD > SCZ	*t*_(25.0)_ = 4.075, *p* = 0.001
lh_inferiortemporal_volume	*F*_(2.0, 36.0)_ = 0.609, *p* = 0.008	HV > SCZ	*t*_(25.0)_ = 4.872, *p* <0.001
lh_lateralorbitofrontal_volume	*F*_(2.0, 36.0)_ = 0.868, *p* = 0.008	HV > SCZ	*t*_(25.0)_ = 4.545, *p* <0.001
		BD > SCZ	*t*_(27.0)_ = 3.03, *p =* 0.016
lh_posteriorcingulate_volume	*F*_(2.0, 36.0)_ = 0.001, *p =* 0.045	HV > SCZ	*t*_(25.0)_ = 3.404, *p =* 0.007
lh_precuneus_volume	*F*_(2.0, 36.0)_ =0.007, *p =* 0.006	HV > SCZ	*t*_(25.0)_ = 4.103, *p =* 0.001
lh_superiorfrontal_volume	*F*_(2.0, 36.0)_ = 0.013, *p =* 0.006	HV > SCZ	*t*_(27.0)_ = 3.868, *p =* 0.002
		BD > SCZ	*t*_(25.0)_ = 4.386, *p =* 0.001
lh_supramarginal_volume	*F*_(2.0, 36.0)_ = 0.028, *p =* 0.013	HV > SCZ	*t*_(25.0)_ = 3.109, *p =* 0.014
rh_entorhinal_volume	*F*_(2.0, 36.0)_ = 0.002, *p =* 0.044	HV > SCZ	*t*_(25.0)_ = 4.388, *p =* 0.001
rh_fusiform_volume	*F*_(2.0, 36.0)_ = 1.42,*p =* 0.036	HV > SCZ	*t*_(25.0)_ = 3.588, *p =* 0.004
rh_inferiorparietal_volume	*F*_(2.0, 36.0)_ = 0.151, *p =* 0.020	HV > SCZ	*t*_(27.0)_ = 3.796, *p =* 0.002
		BD > SCZ	*t*_(25.0)_ = 4.683, *p* <0.001
rh_inferiortemporal_volume	*F*_(2.0, 36.0)_ = 0.562, *p =* 0.004	HV > SCZ	*t*_(25.0)_ = 4.917, p <0.001
rh_lateralorbitofrontal_volume	*F*_(2.0, 36.0)_ = 0.598, *p =* 0.006	HV > SCZ	*t*_(25.0)_ = 4.227, *p =* 0.001
		BD > SCZ	*t*_(27.0)_ = 3.08,*p =* 0.014
rh_medialorbitofrontal_volume	*F*_(2.0, 36.0)_ = 0.005, *p =* 0.025	HV > SCZ	*t*_(25.0)_ = 3.236, *p =* 0.010
rh_precuneus_volume	*F*_(2.0, 36.0)_ = 0.372, *p =* 0.005	HV > SCZ	*t*_(25.0)_ = 4.177, *p =* 0.001
rh_superiorfrontal_volume	*F*_(2.0, 36.0)_ = 0.705, *p =* 0.006	HV > SCZ	*t*_(27.0)_ = 4.24, *p =* 0.001
		BD > SCZ	*t*_(25.0)_ = 3.372, *p =* 0.007
rh_supramarginal_volume	*F*_(2.0, 36.0)_ = 0.017, *p =* 0.017	HV > SCZ	*t*_(25.0)_ = 5.021, *p* <0.001

Results from ANOVA (including genotype) and post-hoc t-test are presented. All significant differences were consistent with the BD > HV > SCZ relationship.

### N-back performance

For both the fMRI and fMRS n-back tasks, SCZ had significantly lower mean response accuracy and longer response times compared to HV and BD groups for the 0-back (*p* < 0.01; *p* < 0.05). For the fMRI task, SCZ had lower mean response accuracy than BD for the 2-back condition (*p* < 0.01), whereas in the fMRS task, SCZ had lower mean response accuracy than both HV and BD (*p* < 0.001; *p* < 0.05). For the fMRS task, SCZ had longer response times than HV (*p* < 0.05). There was no effect of SLC30A3 genotype on fMRI n-back performance.

### FMRI

For 2-back > 0-back contrast, only group (*F*_(16,52)_ = 1.880, *p* = 0.045, Pillai's trace = 0.733, partial η2 = 0.367) was significant, with activation showing the pattern BD > HC > SCZ in all but right dorsolateral prefrontal cortex which showed the activation pattern BD > SCZ > HC.

### ^1^H-MRS

**Figure 2:**
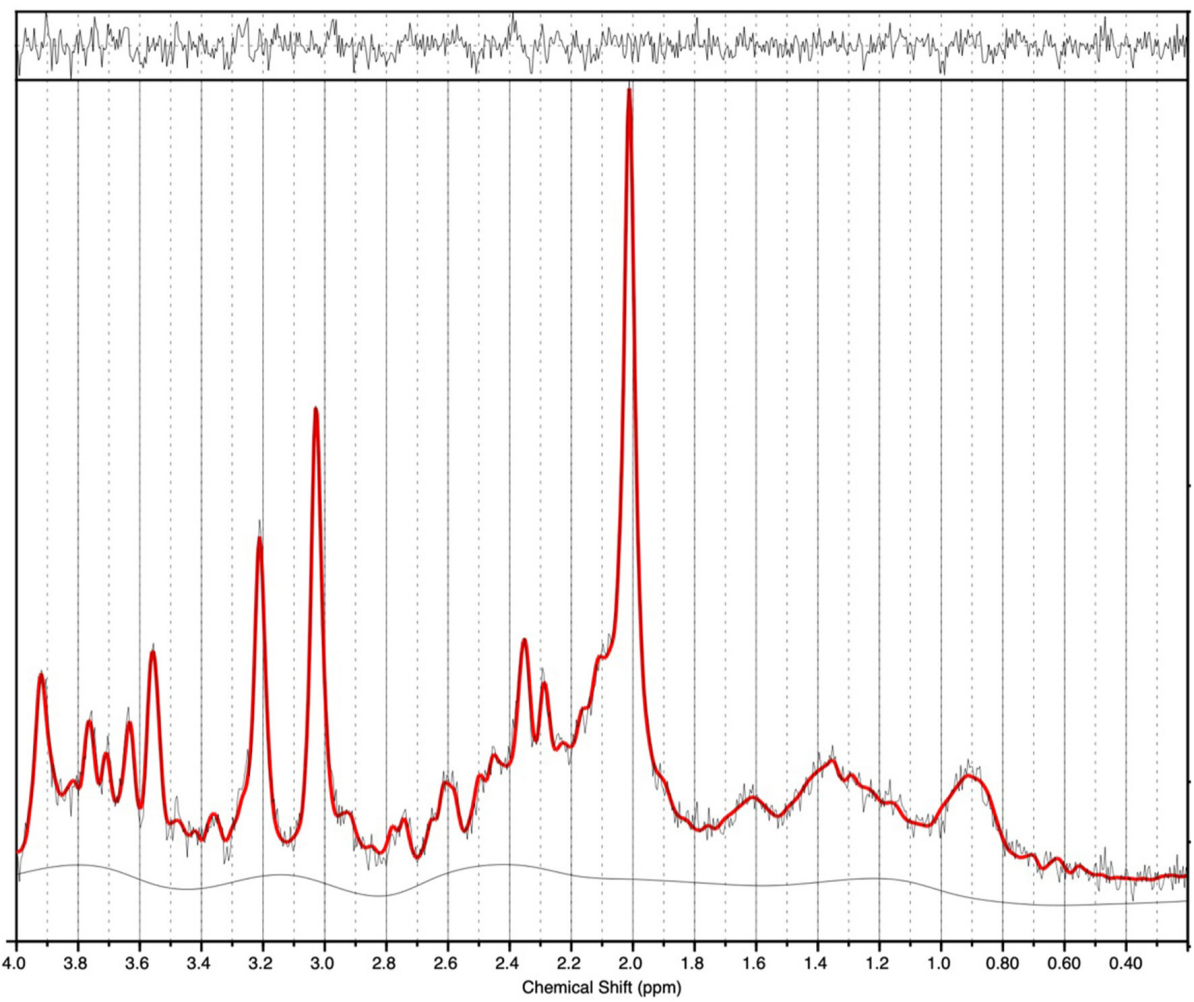
^1^H-MRS spectra (for a single subject). LCModel output is shown with output of the fit (red) overlaid on the acquired spectrum (black). The estimated baseline is displayed under the spectrum in black. For this single subject example, a metabolite FWHM of 0.033 ppm and SNR of 33 was achieved.

^1^H-MRS spectra were generally of a good quality (Figure 2). Data from 1 participant (SCZ) were excluded from analysis due to CV > 20% and SNR < 5. There were no significant main effects of group or genotype or group by genotype interactions for either Glu or Glx in the anterior cingulate cortex. *Post-hoc* testing revealed that BD with at least one copy of the minor allele (*n* = 10) had significantly lower Glu levels compared to those with two copies of the major allele (*n* = 5; *p* = 0.024).

### ^1^H-FMRS

**Figure 3:**
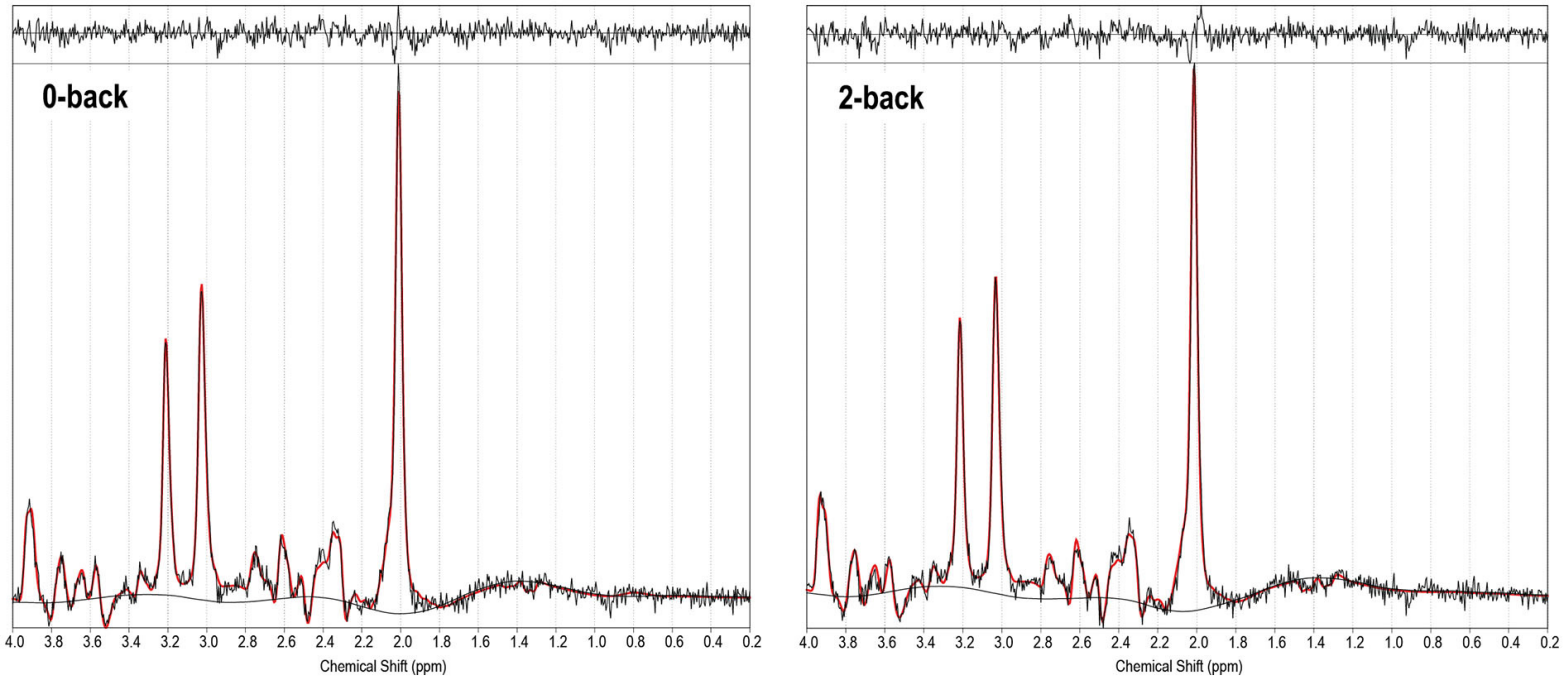
^1^H-fMRS spectra (for a single subject). TARQUIN output is shown for 0-back and 2-back conditions with output of the fit (red) overlaid on the acquired spectrum (black). The estimated baseline is displayed under each spectrum in black. For this single subject example, for the 0-back condition a metabolite FWHM of 0.038 ppm, unsuppressed water FWHM of 0.042 ppm and SNR of 38.9 were achieved, and for the 2-back condition a metabolite FWHM of 0.036 ppm, unsuppressed water FWHM of 0.042 ppm and SNR of 36.6 were achieved.

**Figure 4:**
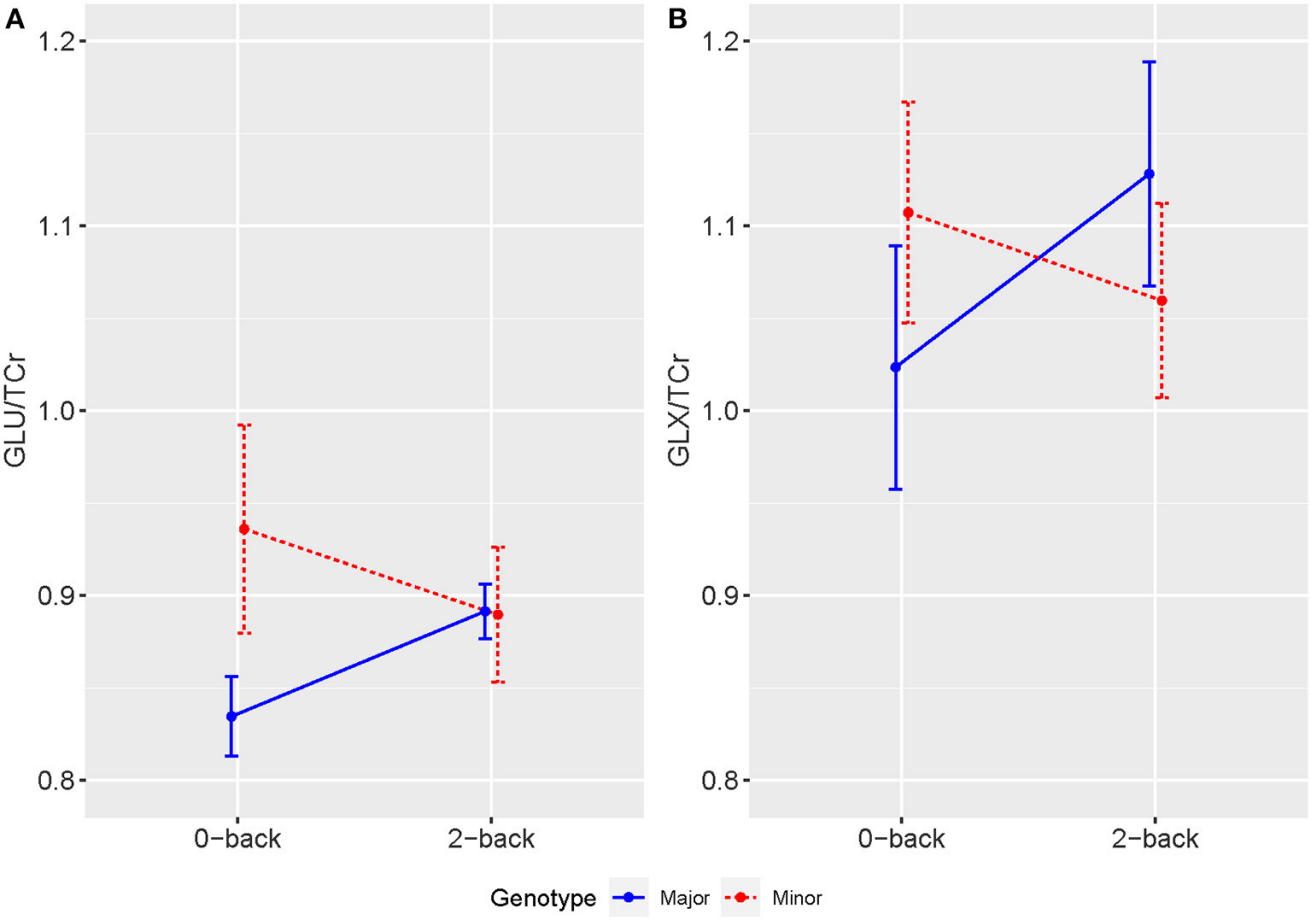
Averaged levels of **(A)** Glu/TCr and **(B)** Glx/TCr in Institutional Units (IU) as measured by ^1^H-fMRS across 0-back and 2-back conditions for individuals with SCZ by genotype. Error bars show SEM.

^1^H-fMRS spectra were generally of a good quality (Figure 3). Data from 1 participant (SCZ) were excluded from analysis due to CV > 20% and SNR < 5. 2x2 rmANOVAs determined a significant n-back condition by genotype interaction for Glu/TCr, *F*_(1,12)_ = 6.407, (*P* = 0.026) and Glx/TCr, *F*_(1,12)_ = 7.878, (*P* = 0.016) in SCZ. In SCZ with the major allele, levels of Glu/TCr and Glx/TCr increased between task conditions, while in the minor allele group levels of both metabolites decreased (Figure 4). In HV and BD there was no significant n-back condition by genotype interaction for either Glu/TCr, or Glx/TCr.

## Discussion

This is the first study to demonstrate an effect of SLC30A3 genotype on brain activity in patients with SCZ and BD type 2. We found that during the n-back task, the minor allele had the effect of reducing activation regardless of diagnosis. We also found that the minor allele was associated with reduced anterior cingulate glutamate concentrations in patients with BD type 2, and that during the n-back task, patients with SCZ who had the minor allele showed reductions in Glu/TCr and Glx/TCr, whereas those with the major allele showed the same increase in Glu/TCr and Glx/TCr seen in healthy volunteers (17). Taken together, these findings support the hypothesis that ZnT3 modulates glutamatergic neurotransmission, and that carrying the minor allele may lead to changes in brain activity and glutamatergic neurotransmission in SCZ and BD type 2. The findings also add support to the hypothesis that glutamatergic abnormalities may be involved in the pathophysiology of schizophrenia (29).

Regarding our ^1^H-MRS data, we found that minor ZnT3 allele carriers in the BD group had significantly lower Glu levels compared to those carrying major ZnT3 alleles. Previous ^1^H-MRS studies provided evidence that glutamate dysfunction plays an important role in the pathophysiology of bipolar disorder (30). While studies have suggested medial prefrontal glutamate levels to be elevated (31, 32), this has not been consistently seen (33, 34), and may be due differences in mood phases, subtypes of bipolar and medication status between studies. Our finding of reduced ACC ^1^H-MRS Glu levels in BD type II minor ZnT3 allele carriers compared to major carriers suggests that variants in ZnT3 encoding gene may play a role in contributing to altered glutamate function in this disorder. Further work using larger sample sizes, examining ZnT3 genotype in BD types 1 and 2 and how this relates to glutamate level measures, symptom burden and treatment-response would be valuable.

For the ^1^H-fMRS data, we found a significant n-back condition by genotype interaction for Glu/TCr and Glx/TCr in the SCZ group. While SCZ with the major ZnT3 alleles showed a general increase in levels of Glu/TCr and Glx/TCr during the 2-back condition compared with the 0-back, those individuals with the minor alleles showed the opposite pattern, with levels reducing between task conditions. We previously reported ^1^H-fMRS n-back findings from this sample (prior to ZnT3 genotyping analysis) (17). Considering individual groups, regardless of ZnT3 genotype, we found that while healthy volunteers showed significant increases in Glu/TCr and Glx/TCr with increasing task difficulty (between the averaged last spectra of the 0-back and the first of the 2-back task conditions), this was not seen in patients with SCZ or BD (17). In the present study, exploring the effects of variants in ZnT3 encoding gene, SCZ with the major genotype showed a general increase in ^1^H-fMRS Glu/TCr and Glx/TCr in the more cognitively demanding 2-back compared to the 0-back condition, which is reflective of the pattern reported in HV (17). In contrast, SCZ with minor ZnT3 genotype showed a general reduction in ^1^H-fMRS glutamate measures between task conditions. These findings are comparable with a previous ^1^H-fMRS study that measured task-related changes in dynamic glutamatergic concentrations in SCZ and healthy volunteers during the performance of a color-word Stroop task at 7 Tesla (35). While healthy volunteers showed significant increases in glutamate concentrations during the Stroop task this was not seen in the schizophrenia group (35). This suggests that SCZ may be associated with blunted activation of dynamic glutamate responses in the ACC to task requirements and considering this present study's findings, abnormal variants in the ZnT3 encoding gene may contribute to this abnormality.

This study has a number of limitations. The sample size is small and so any findings must be seen as preliminary. There are also potential covariates such as age, gender and levels of plasma zinc for which we could not correct. Furthermore, with the ^1^H-fMRS acquisition, The TE of 105 ms was chosen based on a previous glutamate ^1^H-fMRS study (36), however, this TE is quite long with significant J-modulation and attenuation due to T2 relaxation and therefore may not have been most optimal. Future ^1^H-fMRS work should directly compare long and short TE times to assess reliability of glutamate/glutamine/Glx measures.

## Conclusions

This is the first study to demonstrate a functional difference in brain activity and brain glutamate in carriers of the minor SLC30A3 allele in patients with BD and SCZ. It supports the hypothesis that ZnT3 may play a modulatory role in glutamatergic neurotransmission, with relevance to patients with mental illness. Further work is required to determine whether modulation of ZnT3 function may have a therapeutic benefit in these conditions.

## Data availability statement

The original contributions presented in the study are included in the article/supplementary material, further inquiries can be directed to the corresponding author.

## Ethics statement

The studies involving human participants were reviewed and approved by London Harrow Research Ethics Committee. The patients/participants provided their written informed consent to participate in this study.

## Author contributions

JS and JD originated the idea for the study. JS designed the study and wrote the protocol. SK and LJ collected the data. AM and XZ performed the genetic analysis. LJ, MG, AM, XZ, DL, and JS analyzed the data. LJ, MG, SK, AM, XZ, DL, AY, and JS reviewed, edited, and approved the final version of the paper. All authors contributed to the article and approved the submitted version.

## Funding

This research was funded in part by the National Institute for Health Research (NIHR) Biomedical Research Centre at South London and Maudsley NHS Foundation Trust and King's College London. LJ is a Medical Research Council (MRC) Clinical Research Training Fellow (MR/T028084/1). Neither funding source had any role in study design, in the collection, analysis or interpretation of data, in the writing of the report, or in the decision to submit the article for publication.

## Conflict of interest

In the last 3 years, JS has been principle investigator or sub-investigator on studies sponsored by Takeda, Janssen and Lundbeck Plc. He has attended an Investigators' meeting run by Allergan Plc. AY has given paid lectures and attended advisory boards for the following companies with drugs used in affective and related disorders: Astrazenaca, Eli Lilly, Lundbeck, Sunovion, Servier, Livanova, Janssen, Allegan, Bionomics, Sumitomo Dainippon Pharma. He is also a consultant to Johnson & Johnson and to Livanova. The remaining authors declare that the research was conducted in the absence of any commercial or financial relationships that could be construed as a potential conflict of interest.

## Publisher's note

All claims expressed in this article are solely those of the authors and do not necessarily represent those of their affiliated organizations, or those of the publisher, the editors and the reviewers. Any product that may be evaluated in this article, or claim that may be made by its manufacturer, is not guaranteed or endorsed by the publisher.
